# ASSOCIATIONS OF URINARY CONDITIONS WITH VAGINAL MICROBIOTA AND METABOLITES ACROSS REPRODUCTIVE STAGES

**DOI:** 10.1093/geroni/igad104.1438

**Published:** 2023-12-21

**Authors:** Michelle Shardell, Sarah Robbins, Joanna-Lynn Borgogna, Christina Stennett, Carl Yeoman, Anne Burke, Jacques Ravel, Rebecca Brotman

**Affiliations:** University of Maryland School of Medicine, Baltimore, Maryland, United States; University of Maryland School of Medicine, Baltimore, Maryland, United States; Montana State University, Bozeman, Montana, United States; University of Maryland School of Medicine, Baltimore, Maryland, United States; Montana State University, Bozeman, Montana, United States; Gynecology and Obstetrics, Baltimore, Maryland, United States; University of Maryland School of Medicine, Baltimore, Maryland, United States; University of Maryland School of Medicine, Baltimore, Maryland, United States

## Abstract

The genitourinary syndrome of menopause describes signs/symptoms of the female genitourinary tract, including urinary symptoms/conditions, resulting from declining estrogen in menopause. Menopause is also associated with declining abundance of Lactobacillus spp. in the vagina. Vaginal and urinary microbiota are highly concordant, where low abundance of certain Lactobacillus spp. have demonstrated associations with urinary symptoms/conditions. The vaginal microenvironment also includes microbially- and host-produced metabolites that may impact genitourinary health. Thus, we aimed to identify vaginal microbial and metabolic signatures of urinary conditions. In a two-year cohort study comprising 476 participants aged 35-60 years who contributed 1,153 semiannual person-visits, 15.2% (27/178) postmenopausal and 10.4% (31/298) non-postmenopausal participants self-reported a history of urinary conditions (overactive bladder, recurrent urinary tract infection[UTI], or cystitis). Among postmenopausal participants, history of urinary conditions was associated with higher covariate-adjusted odds of L. gasseri-dominated (OR[Odds Ratio]=4.29, 95% Confidence Interval[CI]=1.31-14.88) versus L. crispatus-dominated communities. Significant metabolites were enriched for the lipids sphingomyelins and metabolites involved in glycolysis (P<.05). Urinary conditions were associated with joint microbiota-metabolite profiles characterized by higher sphingomyelins, pyruvate, and genera Varibaculum and Escherichia; and lower dihydroxyacetone phosphate, nicotinamide, Gardnerella vaginalis, and genus Fusobacterium (P<.001). In contrast, among non-postmenopausal participants, urinary conditions did not significantly relate to microbial community (P>.10); however, sphingomyelins remained significantly enriched (P<.05). Signatures among postmenopausal participants include known UTI pathogen E. coli and metabolites that may facilitate and fuel its growth. Among non-postmenopausal participants, urinary conditions more strongly related to metabolic profiles than microbiota. Investigation of these signatures in incident/recurrent urinary conditions is needed.

